# Systemic Vascular Calcification After Lung Transplantation: The Role of Mammographic Breast Arterial Calcifications

**DOI:** 10.3390/jcm14238579

**Published:** 2025-12-03

**Authors:** Jonathan Andreas Saenger, Jasmin Happe, Elizabet Nikolova, Caroline Maier, Ela Uenal, Denise Bos, Thomas Frauenfelder, Andreas Boss

**Affiliations:** 1 Diagnostic and Interventional Radiology, University Hospital Zurich, University Zurich, 8091 Zurich, Switzerlandelizabet.nikolova@usz.ch (E.N.);; 2 Institute of Diagnostic and Interventional Radiology, GZO Regional Health Center, 8620 Wetzikon, Switzerland; 3 Institute of Diagnostic and Interventional Radiology and Neuroradiology, University Hospital Essen, University Duisburg-Essen, 45147 Essen, Germany

**Keywords:** lung transplantation, vascular calcification, screening, mammography

## Abstract

**Objective:** This study systematically evaluates the association between breast arterial calcifications (BACs) on mammography and systemic vascular calcifications detected by computed tomography (CT) in female lung transplant recipients, representing the first investigation of this relationship in this population. **Methods:** This retrospective single-center study included 78 female LTX recipients who underwent both digital mammography and thoracoabdominal CT. BACs were graded on a four-point scale. Calcifications in seven arterial territories were visually assessed on CT with a binary scale (“present”, “not present”) and graded on a severity scale of systemic calcifications ranging from no involvement (0) and minor vascular calcification (1–4 vessels) to major vascular calcification (5–10 vessels). Associations between BACs and systemic vascular calcifications were analyzed using correlation, logistic regression, and ROC analysis. **Results:** BACs were present in 31 of 78 patients (40%). Vascular calcifications were frequent, most often affecting the thoracic aorta (82%), abdominal aorta (76%), and iliac arteries (72–73%). Coronary calcifications were more common in BAC-positive than BAC-negative patients (77% vs. 40%, *p* = 0.002). BAC grade correlated with the cumulative vascular calcification (ρ = 0.35, *p* = 0.0017). In ROC analysis, BACs discriminated patients with vascular calcification with fair accuracy (AUC 0.73, 95% CI 0.67–0.79). In multivariable logistic regression adjusted for age, diabetes, chronic kidney disease, and hypertension, BACs remained independently associated with systemic calcification, with an adjusted odds ratio of approximately 2.0 for ≥1 affected vascular territory. Interreader agreement for the 4-level BAC score was excellent with a quadratic-weighted κ of 0.91 and a linear-weighted κ of 0.88. **Conclusions:** BACs are common in female LTX recipients and independently predict systemic vascular calcifications. Their detection in routine mammography may provide a simple, opportunistic marker to support cardiovascular risk stratification in this high-risk population.

## 1. Introduction

Lung transplantation is a recognized treatment for individuals with end-stage lung disease, leading to substantial improvement in quality of life and survival rates. Advancements in surgical methods, immunosuppressive regimens, and perioperative management have improved short-term results; yet, long-term survival remains limited [1]. Chronic allograft malfunction, infection, cancer, and cardiovascular disease are major factors contributing to late morbidity and mortality in this population [2,3,4].

Cardiovascular complications significantly influence long-term survival following lung transplantation [5]. The pathophysiological interaction between advanced lung disease and vascular calcification is not fully understood, but both share risk factors, including age, diabetes, hypertension, and postmenopausal hormone fluctuations [6]. For patients after lung transplantation, immunosuppressive therapy is essential for graft preservation; however, it may accelerate vascular calcification through the induction of endothelial dysfunction and the promotion of unfavorable metabolic profiles [3]. Calcineurin inhibitors and corticosteroids play a major role in damaging blood vessels and starting early arterial calcification [7,8]. Statins and vitamin D supplements, often used post-transplant, may also affect vascular calcification through their effects on lipid metabolism and mineral balance. Arterial calcification results from disturbed calcium metabolism and inflammation, with lipid deposition, endothelial dysfunction, and foam-cell-driven plaque formation progressing across vascular beds [9,10,11,12,13,14,15]. Systemic arterial calcifications in the thoracic and abdominal aorta, iliac, and renal arteries are frequently found and associated with adverse outcomes, including increased post-transplant mortality [16]. Early recognition of these changes is therefore critical for improving risk stratification and guiding preventive strategies.

For cardiovascular risk assessment traditional non-invasive tests, including dobutamine stress echocardiography (DSE), myocardial perfusion imaging (MPI), stress cardiac magnetic-resonance imaging, and cardiac CT, offer valuable information but have limited sensitivity and specificity in this patient group [3]. While positron emission tomography (PET) demonstrates higher accuracy, invasive coronary angiography remains the reference standard in many centers, particularly for older recipients [3]. Prior studies demonstrated a correlation between BAC and coronary artery calcification (CAC), aortic calcifications, and vascular events in the general female population [15,17,18,19]. Recent work has shown that BAC is prevalent in female lung transplant recipients, yet its relationship with systemic vascular calcifications has not been systematically investigated.

This study is aimed at investigating the prevalence of systemic vascular calcification following lung transplantation in female recipients, to investigate the correlation between breast arterial calcifications and vascular calcifications on CT, and to assess the diagnostic efficacy of BAC as a potential screening tool.

## 2. Materials and Methods

### 2.1. Patient Selection

The local ethics committee approved this retrospective study on 6 July 2021 (Nr. 2021-01095). All procedures were performed in compliance with institutional guidelines, federal regulations, and the principles of the Declaration of Helsinki. The study cohort included adult female patients who underwent lung transplantation at our institution and received at least one post-transplantation mammography and at least one thoracoabdominal CT scan. Patients with concurrent transplantations, such as kidney or liver, were excluded. Clinical data, including relevant comorbidities such as chronic kidney disease, diabetes mellitus, and pulmonary hypertension or hypertension, were extracted from electronic medical records.

### 2.2. Mammography Protocol and Assessment

Standard craniocaudal and mediolateral oblique projections were obtained for each breast [20]. Digital breast tomosynthesis was not available for all patients and was thus excluded from this analysis. Mammography was performed using one of the following systems: Senographe Essential Acquisition System ADS 56.21.3 (General Electric, Boston, MA, USA), Image SDL or Class System (Internazionale Medico Scientifica/Giotto, Sasso Marconi, Italy), Selenia Dimensions (Hologic, Marlborough, MA, USA), or Mammomat Novation DR/Mammomat Inspiration System (Siemens Healthcare GmbH, Forchheim, Germany). Breast density and BI-RADS classification were systematically assessed [21]. BACs were defined as linear or tubular calcifications aligned with the expected vessel trajectory, often exhibiting a characteristic parallel “railroad track” appearance [21]. To assess the extent of BAC, a structured visual scoring system consisting of four grades was applied: BAC Grade 1 included patients with no visible calcifications or only sporadic scattered microcalcifications. BAC Grade 2 was defined as a few scattered, discontinuous calcifications within a single region, with uncertain association to a tubular structure and resembling fibroglandular microcalcifications. Calcifications clearly originating from BAC but limited in extent were classified as BAC Grade 3, while BAC Grade 4 represented extensive or pronounced BAC [17].

### 2.3. CT Protocol

Routinely performed thoracoabdominal CT scans were identified through the institutional Picture Archiving and Communication System (PACS). CT scans were performed on a second-generation dual-source CT system (Siemens Somatom Definition Flash), a third-generation dual-source CT system (Siemens Somatom Force), or a first-generation dual-source photon-counting detector CT system (Siemens NAEOTOM Alpha), all from Siemens Healthcare GmbH (Forchheim, Germany). The following vascular territories were assessed: thoracic and abdominal aorta, bilateral common iliac and carotid arteries, bilateral renal arteries, and the superior mesenteric artery. Each was classified as “calcification present” or “absent.” Systemic calcification severity was graded by counting affected territories (0–10) and categorized as no involvement (0), minor (1–4 vessels), or major (5–10 vessels) according to a modified Shigemura et al. scale [17]. The presence of CAC was classified as “calcification present” or “absent.” For the vast majority of patients, dedicated heart CTs or non-contrast thoracic CTs were unavailable, preventing the calculation of the Agatson score for classification of coronary sclerosis.

### 2.4. Reader Protocol

One radiologist (J.A.S., 4 years of experience) reviewed all mammograms and CT scans. A blinded second reader (J.H., 3 years) independently assessed a random 25% sample.

### 2.5. Statistical Analysis

All statistical analyses were conducted using R (version 4.3.2, R Foundation for Statistical Computing, Vienna, Austria). Continuous variables are reported as median with interquartile range (IQR) or mean with standard deviation (SD), as appropriate; categorical variables are presented as absolute numbers and percentages. Descriptive analyses were conducted to determine the prevalence of BACs on mammography and vascular calcifications on CT across predefined territories. Associations between BAC and the presence of vascular calcification in individual CT territories were tested using Pearson’s chi-square test or Fisher’s exact test, depending on expected frequencies. Correlation between the BAC visual score and the cumulative CT vascular calcifications (range 0–10) was assessed using Spearman’s rank correlation coefficient. To assess the diagnostic performance of BAC in identifying systemic calcification, a receiver operating characteristic (ROC) curve analysis was performed using the presence of ≥1 calcified vascular region on CT as the reference standard. The area under the curve (AUC) with 95% confidence intervals was reported. To assess the diagnostic performance of BAC in identifying the prevalence of calcified plaques in different body regions, univariable logistic regression models were fitted for each vascular territory using BAC presence as the predictor. Systemic vascular calcification was defined as ≥1 calcified vascular territory on CT. Firth’s penalized logistic regression analysis was used to determine whether BAC presence independently predicted systemic vascular calcification, adjusting for age, diabetes mellitus, chronic kidney disease, pulmonary arterial hypertension, and arterial hypertension. Odds ratios (ORs) with 95% confidence intervals (CIs) and corresponding *p*-values were reported; statistical significance was defined as a two-sided *p*-value less than 0.05. Interobserver agreement for BAC and vasosclerosis scoring on CT was evaluated using Cohen’s kappa, interpreted according to Landis and Koch: values ≤ 0.20 indicated poor agreement, 0.21–0.40 fair, 0.41–0.60 moderate, 0.61–0.80 substantial, and 0.81–1.00 excellent agreement.

## 3. Results

### 3.1. Patient Cohort

**Figure 1:**
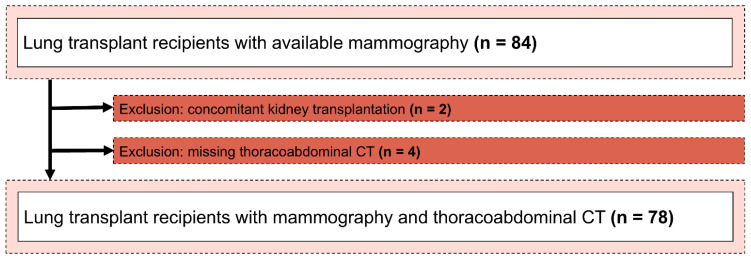
Flowchart illustrating inclusion and exclusion.

A total of 78 female lung transplant recipients met the inclusion criteria (Figure 1). The median age at mammography was 61 years (IQR, 53–66). Thirty-one patients (40%) were either current or past smokers, with a median smoking exposure of 14 pack-years (IQR, 0–25). Chronic obstructive pulmonary disease (COPD) was the predominant underlying diagnosis (44%), succeeded by cystic fibrosis (15%), idiopathic pulmonary fibrosis and lymphangioleiomyomatosis (each 7%), alpha-1 antitrypsin deficiency (6%), and pulmonary arterial hypertension (4%). Other disorders, such as non-CF bronchiectasis, sarcoidosis, and interstitial lung disease, were infrequent (<2% each). Patient comorbidities included chronic kidney disease (47%), arterial hypertension (31%), and diabetes mellitus (9%) (Table 1).

### 3.2. Mammographic Assessment

**Figure 2:**
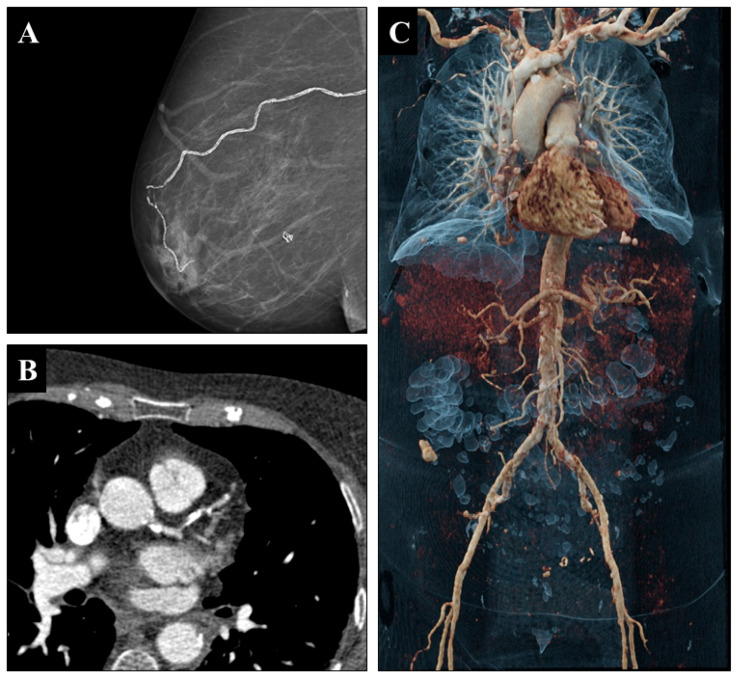
Screening mammography in a female patient in her late fifties, performed approximately eight years after bilateral lung transplantation, demonstrates severe breast arterial calcifications in the right breast on the MLO projection Image (**A**). Coronary CT angiography performed about 2 years later shows severe calcifications in left anterior descending artery and the left circumflex artery Image (**B**). A three-dimensional cinematic rendering of a CT scan performed in the same year depicts the aorta and major branches with advanced systemic vascular calcifications Image (**C**).

BACs were identified in 31 patients (40%). The median age was identical for patients with and without BACs (61 years vs. 61 years, *p* = 0.73). The interval from lung transplantation to mammography was significantly longer in patients with BAC compared to without BAC (8 years vs. 4 years, *p* = 0.001). Breast density was ACR a in 8%, ACR b in 32%, ACR c in 47%, and ACR d in 13%. The majority of mammograms were categorized as BI-RADS 2 (*n* = 74; 86%), while fewer were classified as BI-RADS 3 (*n* = 7; 8%) or BI-RADS 4 (*n* = 3; 4%). Neither breast density (*p* = 0.61) nor BI-RADS assessment (*p* = 0.56) differed significantly between women with and without BAC. The distribution of BAC grades showed that 45 patients (60%) exhibited no or only sporadic microcalcifications (grade 1), 5 patients (6%) were classified as grade 2 with mild discontinuous vascular calcifications, 14 patients (12%) were categorized as grade 3 with more pronounced yet limited BAC, and 14 patients (13%) were assessed as grade 4 with significant, confluent calcifications. Analysis of baseline characteristics indicated no significant disparities in smoking history, diabetes, or chronic renal disease between BAC-positive and BAC-negative patients. A borderline significant difference for elevated arterial hypertension rates was seen in BAC-positive patients (35% vs. 27%, *p* = 0.05). Figure 2 illustrates a representative case with severe BAC accompanied by systemic vascular calcification.

### 3.3. CT Assessment

The median interval between mammography and CT was 26 days (IQR, −101 to 203), indicating that in multiple cases, CT was performed prior to mammography. Given the chronic nature of vascular calcification, differences in timing between CT and mammography (median interval 26 days, range −101 to 203) were unlikely to affect results. Systemic vascular calcifications were prevalent in various territories (Table 2). The thoracic aorta was most affected (82%), followed by the abdominal aorta at 76% and the iliac arteries at 72–73%. About 55% of patients had coronary calcifications with a significantly higher prevalence in BAC-positive patients compared to BAC-negative patients (77% vs. 40%, *p* = 0.002). Renal artery calcifications were seen in 44% of cases overall, with a tendency for increased prevalence in BAC-positive patients (58% compared to 34%, *p* = 0.06). Carotid calcifications were less common and occurred in 14% of cases on the left side and 12% on the right side. The superior mesenteric artery was involved in 21% of the cases.

### 3.4. Correlation and Diagnostic Accuracy

BAC grade correlated moderately with the cumulative CT vascular calcification (Spearman ρ = 0.35, *p* = 0.0017; *n* = 78). Receiver operating characteristic analysis showed that the presence of BAC discriminated patients with vascular calcification with fair accuracy, with an area under the curve (AUC) of 0.73 (95% CI 0.67–0.79).

### 3.5. Regression Analyses

**Figure 3:**
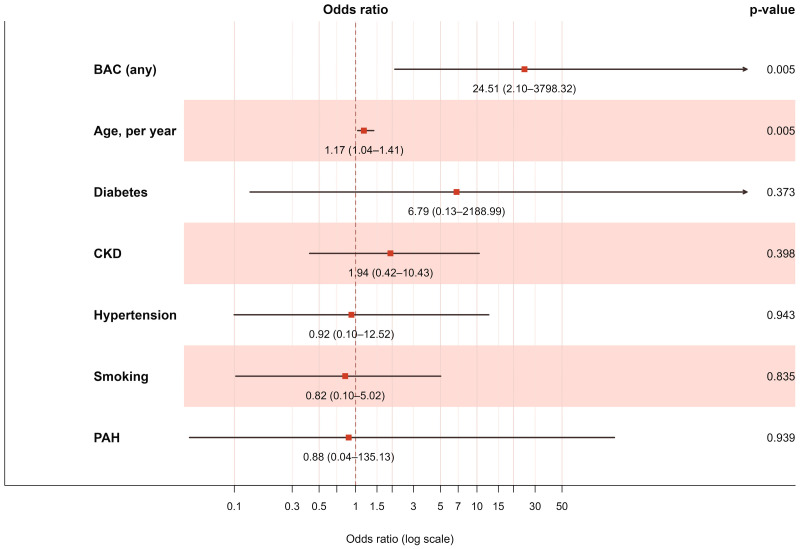
Firth’s penalized logistic regression model for the presence of systemic vascular calcification on CT (≥1 calcified vascular territory). Penalization was applied to account for quasi-complete separation. Odds ratios with 95% confidence intervals are plotted on a log scale. Arrows indicate clipped intervals; red dashed line marks OR = 1.

**Figure 4:**
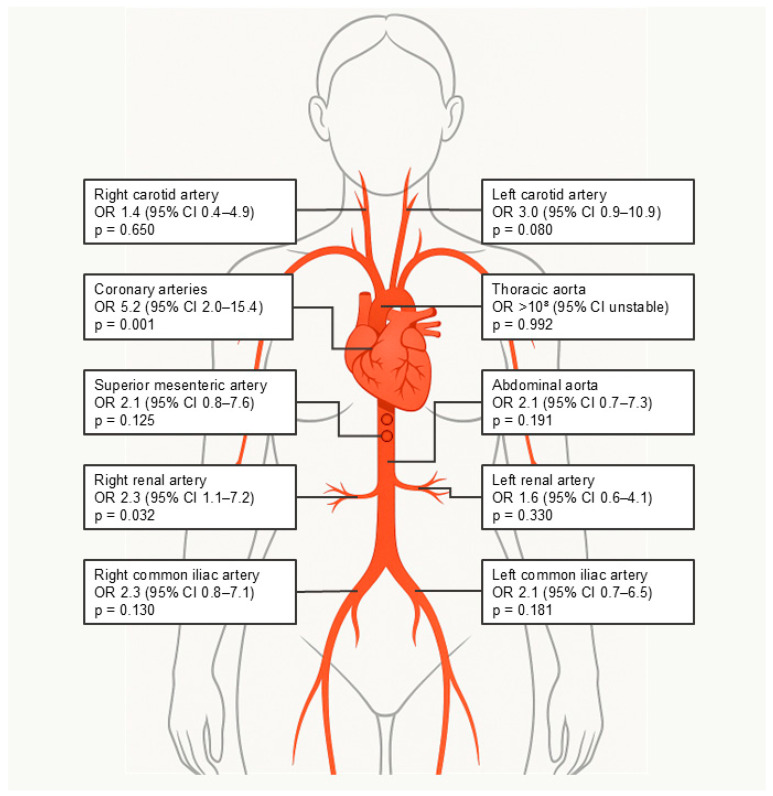
Illustration of the prevalence of calcified plaques in different body regions. Values for the thoracic aorta reflect model convergence failure due to complete separation.

In multivariable Firth logistic regression adjusted for age, diabetes, chronic kidney disease, and hypertension, the presence of BAC remained an independent predictor of systemic vascular calcification, approximately doubling the odds of ≥1 affected vascular territory (Table 3, Figure 3). Univariable analyses demonstrated that BAC was significantly associated with coronary artery calcifications (77% vs. 40%, *p* = 0.002) and showed the strongest odds ratios for the coronary arteries and abdominal aorta. For the thoracic aorta, univariable analysis was not possible to calculate due to complete separation of the data, as all patients with BAC had calcification in this territory (Figure 4).

### 3.6. Interobserver Agreement

Inter-reader agreement was excellent for the BAC assessment, with a quadratic-weighted κ of 0.91 and a linear-weighted κ of 0.88, indicating near-perfect consistency. For the systemic vascular calcification, agreement was more moderate, with a quadratic-weighted κ of 0.54 and a linear-weighted κ of 0.66 between, showing a modest systematic variation in score assignment between versions.

## 4. Discussion

Our research shows that BAC is common among female lung transplant recipients, with approximately 40% exhibiting BAC on mammography. BAC correlated with systemic vascular calcifications detected on CT and remained an independent predictor in multivariable analyses. While the strongest associations were observed for the coronary arteries, similar trends were evident across other vascular territories but did not reach statistical significance, likely due to the limited sample size. These findings suggest that BAC may serve as a practical marker for identifying patients with systemic vascular calcification.

While earlier studies primarily focused on the relationship between mammographically detected BAC and CAC, our study is the first to extend this association to systemic vascular calcification and cardiovascular risk in lung transplant recipients. Our results align with previous research demonstrating that vascular calcification is a frequent comorbidity in lung transplant recipients, with a prevalence of 86% in our cohort. Previous research indicated that up to 100% of patients exhibit vascular calcification post-transplantation, with 44% categorized as having high vascular calcification and 56% as having low calcification [22]. While our sample size limited statistical power for individual vascular territories, a consistent signal emerged across multiple regions, with significant associations for the coronary and right renal arteries and a trend for the left carotid artery. In the absence of comparable data from lung transplant recipients, insights derived from the general population suggests that women with BAC exhibited significantly elevated rates of coronary and aortic calcifications on CT [19]. Although the presence of BAC has been associated with an increased risk of carotid atherosclerosis, as demonstrated by Sedighi et al., it is also linked to a higher risk of stroke, suggesting that these associations may extend to transplant recipients as well [11,23].

Data on the prevalence of CAC in lung transplant recipients remain scarce and difficult to compare, as reported rates vary widely across studies of patients with end-stage lung disease; in our cohort, however, CAC prevalence was markedly higher at 55% compared with 11% in pooled data from transplant candidates in the literature [3]. This variability likely reflects differing stratification approaches and the absence of standardized diagnostic guidelines across centers [3]. Identifying and grading BAC on mammography may aid in detecting patients with underlying vascular calcification, given the well-established association between BAC and coronary artery calcification. A 2020 meta-analysis by Lee et al., including 35,583 patients, found that BAC was associated with CAD, diabetes mellitus, and hypertension, while showing interestingly an inverse association with smoking [24]. As about 33% of our lung transplant recipients reported to be active or former smokers, this might have reduced the prevalence of patients with BAC. Our cohort did not reveal an association between chronic kidney disease, diabetes mellitus, or arterial hypertension; however, these conditions are recognized risk factors for BAC and vascular arteriosclerosis, especially coronary artery disease. BAC primarily impacts the medial layer of the mammary arteries and is strongly associated with diabetes and hypertension [24,25]. Multiple studies indicate that BAC serves as an independent predictor of coronary artery calcification, surpassing traditional risk factors [9,10,11,12,13,14,15]. Additionally, its detection via mammography may exceed individual clinical predictors in identifying women with CAC [18]. Multiple longitudinal studies linked BAC to poor cardiovascular outcomes in the general population and demonstrated an association with increased heart failure, coronary heart disease, and cardiovascular and coronary mortality, regardless of traditional risk factors [11,14]. Schnatz et al. reported that women with BAC exhibited over a threefold increased risk of CHD, while Hendriks et al. identified BAC as a predictor of CHD and combined CVD events in the Prospect-EPIC cohort, noting elevated risk estimates in women with severe BAC [13,15].

In patients following lung transplantation cardiovascular disease significantly contributes to non-pulmonary morbidity and mortality. Almost all recipients exhibit the emergence of at least one new cardiovascular risk factor, such as hypertension, diabetes, or dyslipidemia, during the initial three years, with approximately 40% developing multiple risk factors [26]. Likely due to this risk factor, approximately one-third of late mortality in lung transplant recipients is due to major adverse cardiac events, such as myocardial infarction and heart failure [6]. In addition to these risk factors, chronic vascular injury and inflammation likely contribute to the heightened cardiovascular burden after transplantation [27,28]. Elevated circulating cytokines such as interleukin-6 (IL-6), tumor necrosis factor-α (TNF-α), and interferon-γ (IFN-γ) have been described in affected patients, along with evidence of endothelial activation and microvascular injury extending beyond the transplanted lung [27,28]. These mechanisms mirror the pathways implicated in vascular remodeling and medial arterial calcification, in which chronic inflammation induces oxidative stress, endothelial dysfunction, and osteogenic differentiation of vascular smooth muscle cells [27,28]. Such inflammatory and vascular changes are also observed in Chronic lung allograft dysfunction (CLAD), encompassing bronchiolitis obliterans syndrome (BOS) and restrictive allograft syndrome (RAS) [27,28]. CLAD is increasingly recognized not as a purely pulmonary phenomenon but as a systemic process driven by persistent alloimmune activation, recurrent infection, and chronic inflammation. Registry data show that nearly half of all recipients develop CLAD within five years, and once established, survival is markedly reduced [6,29,30]. These processes might be further amplified by long-term immunosuppression, as recent studies report [31]. Hence, BAC in this population may not only reflect conventional cardiovascular risk but also serve as a subtle imaging biomarker of ongoing systemic inflammatory injury.

This retrospective single-center study has several limitations that may affect generalizability. The limited cohort size reduces statistical power, particularly for analyses involving less common vascular territories. The lung transplant recipients in this study may represent a selected cohort, as only patients who survived long enough to receive mammography were assessed. Secondly, BAC was assessed using a semi-quantitative visual scoring system. Although this method is practical for routine use, it may fail to detect subtle variations in calcification burden. Also, while BAC demonstrated an association with systemic vascular calcifications on CT, the study was not structured to evaluate clinical outcomes. We posited that increased calcification indicates elevated cardiovascular risk, aligning with existing evidence; however, we did not monitor endpoints such as myocardial infarction, stroke, or mortality. The wide interval between mammography and CT and the heterogeneity of CT protocols, including differences in contrast administration, may have affected the assessment of vascular calcifications. The inconsistent coverage of the carotid arteries limited assessment, as the carotid bulb, a common site of vascular calcification, was not uniformly included in all examinations.

Future studies should focus on whether the identification and quantification of BAC can enhance cardiovascular risk stratification and ultimately aid in the prevention of cardiovascular events, both in the general population and in high-risk groups, such as lung transplant recipients. In this context, the detection of BAC on mammography may serve as a practical marker for the need for enhanced cardiovascular assessment and tailored preventive strategies, thereby enabling a more individualized post-transplant management approach. For grade 1 BAC, no further action might be required; for grade 2, biopsy can be considered if microcalcifications are ambiguous, with no need for additional cardiac work-up if BAC is confirmed; for grades 3–4, comprehensive cardiovascular risk assessment might be advisable.

This study shows that breast arterial calcifications on mammography are associated with systemic vascular calcifications in female lung transplant recipients. Their detection may serve as a simple marker to support cardiovascular risk assessment in this population.

## Figures and Tables

**Table 1:** Baseline characteristics of the study population by group (BAC vs. No BAC).

Characteristic	Overall (*n* = 78)	No BAC (*n* = 47)	BAC (*n* = 31)	*p*-Value
Age at mammography (years)	61 (53–66)	61 (53–66)	61 (53–66)	0.730 ^1^
Time since Transplantation (years)	6 (3–10)	4 (2–7)	8 (5–13)	0.001 ^1^
Current or former smoker	31 (33)	21 (43)	10 (22)	0.347 ^2^
Pack years	14 (0–25)	16 (0–30)	11 (0–25)	0.295 ^3^
Chronic kidney disease	45 (47)	25 (51)	20 (44)	0.357 ^2^
Diabetes mellitus	7 (7)	2 (4)	5 (11)	0.107 ^2^
Arterial hypertension	29 (31)	13 (27)	16 (35)	0.054 ^2^
Pulmonary arterial hypertension	6 (6)	3 (6)	3 (7)	0.677 ^2^

Median (Q1, Q3) or *n* (%) are shown unless otherwise specified. Some percentages may not sum to 100% due to rounding. ^1^ Wilcoxon rank sum test. ^2^ Fisher- Exact-Test ^3^ Welch Two Sample *t*-test.

**Table 2:** Vascular characteristics of the study population by group (BAC vs. No BAC).

Characteristic	Overall (*n* = 78)	No BAC (*n* = 47)	BAC (*n* = 31)	*p*-Value ^1^
Right carotid artery	11 (14)	6 (13)	5 (16)	0.746
Left carotid artery	13 (17)	5 (11)	8 (26)	0.120
Coronary arteries	43 (55)	19 (40)	24 (77)	0.002
Thoracic aorta	64 (82)	33 (70)	31 (100)	n/a ^2^
Abdominal aorta	59 (76)	33 (70)	26 (84)	0.191
Superior mesenteric artery	16 (21)	7 (15)	9 (29)	0.158
Right renal artery	34 (44)	16 (34)	18 (58)	0.061
Left renal artery	28 (36)	15 (32)	13 (42)	0.470
Right common iliac artery	56 (72)	31 (66)	25 (81)	0.203
Left common iliac artery	57 (73)	32 (68)	25 (81)	0.299

*n* (%) are shown unless otherwise specified. Some percentages may not sum to 100% due to rounding. ^1^ Fisher- Exact-Test ^2^ For the thoracic aorta, statistical testing was not applicable due to complete separation, as all BAC-positive patients exhibited calcification in this territory.

**Table 3:** Distribution of vascular calcification by breast arterial calcification (BAC) grade.

Scoring Systems	Overall (*n* = 78)	Grade 1 (*n* = 47)	Grade 2 (*n* = 5)	Grade 3 (*n* = 14)	Grade 4 (*n* = 14)	*p*-Value ^1^
Vascular Calcification						0.066
No Vascular Calcification (0)	11 (14)	11 (23)	0 (0)	0 (0)	0 (0)	
Minor Vascular Calcification (1–4)	19 (24)	13 (28)	0 (0)	4 (29)	2 (14)	
Major Vascular Calcification (5–10)	48 (62)	23 (49)	3 (100)	10 (71)	12 (86)	

Values are presented as *n* (%) unless otherwise specified. Percentages may not sum to 100% due to rounding. ^1^ Fisher-Exact-Test.

## Data Availability

The datasets presented in this article are available upon reasonable request.

## Abbreviations

BAC	breast arterial calcification
BI-RADS	Breast Imaging Reporting and Data System
CAC	coronary artery calcification
CT	computed tomography
